# Evaluating the Optimal Management of Inoperable Giant Cell Tumors of the Spine: A Systematic Review and Meta-Analysis

**DOI:** 10.3390/cancers14040937

**Published:** 2022-02-14

**Authors:** Paolo Palmisciano, Gianluca Ferini, Andrew L. Chen, Kishore Balasubramanian, Abdurrahman F. Kharbat, Navraj S. Sagoo, Othman Bin Alamer, Gianluca Scalia, Giuseppe E. Umana, Salah G. Aoun, Ali S. Haider

**Affiliations:** 1 Department of Neurosurgery, Trauma Center, Gamma Knife Center, Cannizzaro Hospital, 95126 Catania, Italy; umana.nch@gmail.com; 2 Department of Radiation Oncology, REM Radioterapia srl, 95029 Viagrande, Italy; gianluca.ferini@grupposamed.com; 3 Texas Tech University Health Sciences Center School of Medicine, Lubbock, TX 79430, USA; andrew.chen@ttuhsc.edu (A.L.C.); ak.kharbat@ttuhsc.edu (A.F.K.); 4 Texas A&M University College of Medicine, Houston, TX 77030, USA; kishore.b@tamu.edu (K.B.); aalam@mdanderson.org (A.S.H.); 5 Department of Orthopaedic Surgery, University of Texas Southwestern Medical Center, Dallas, TX 75390, USA; navraj.sagoo@utsouthwestern.edu; 6 Department of Neurosurgery, King Abdullah International Medical Research Center, Riyadh 11451, Saudi Arabia; oabinalamer@gmail.com; 7 Department of Neurosurgery, Highly Specialized Hospital and of National Importance “Garibaldi”, 95126 Catania, Italy; gianluca.scalia@outlook.it; 8 Department of Neurological Surgery, University of Texas Southwestern Medical Center, Dallas, TX 75390, USA; salah.aoun@utsouthwestern.edu; 9 Department of Neurosurgery, The University of Texas M.D. Anderson Cancer Center, Houston, TX 77030, USA

**Keywords:** denosumab, embolization, giant cell tumor, radiotherapy, spine oncology

## Abstract

**Simple Summary:**

Spine giant cell tumors (SGCTs) are intermediate malignant bone tumors, sometimes aggressive and responsible for debilitating axial pain and sensorimotor impairments. Non-surgical therapies, including denosumab, radiotherapy, and selective arterial embolization (SAE), have shown promising results in the treatment of patients with inoperable SGCTs. In this systematic review, we aimed to comprehensively analyze the current literature on denosumab, radiotherapy, and SAE for inoperable SGCTs, comparing treatment outcomes and complications using a random-effect model meta-analysis. We found that all three treatments were equally effective in providing symptom improvement and radiological tumor response, also showing low and comparable rates of treatment-related complications. Patients treated with denosumab showed lower rates of local recurrences and distant metastases.

**Abstract:**

Background: Surgical resection remains the preferred treatment in spine giant cell tumors (SGCTs), but it is not always feasible. Conservative strategies have been studied for inoperable cases. We systematically reviewed the literature on inoperable SGCTs treated with denosumab, radiotherapy or selective arterial embolization (SAE). Methods: PubMed, Scopus, Web-of-Science, Ovid-EMBASE, and Cochrane were searched following the Preferred Reporting Items for Systematic Reviews and Meta-Analyses (PRISMA) guidelines to include studies of inoperable SGCTs treated with denosumab, radiotherapy or SAE. Treatment outcomes were analyzed and compared with a random-effect model meta-analysis. Results: Among the 17 studies included, 128 patients received denosumab, 59 radiotherapy, and 43 SAE. No significant differences in baseline patient characteristics were found between the three groups. All strategies were equally effective in providing symptom improvement (*p* = 0.187, I^2^ = 0%) and reduction in tumor volume (*p* = 0.738, I^2^ = 56.8%). Rates of treatment-related complications were low (denosumab: 12.5%; radiotherapy: 8.5%; SAE: 18.6%) and comparable (*p* = 0.311, I^2^ = 0%). Patients receiving denosumab had significantly lower rates of local tumor recurrence (10.9%) and distant metastases (0%) compared to patients receiving radiotherapy (30.5%; 8.5%) or SAE (35.6%; 7%) (*p* = 0.003, I^2^ = 32%; *p* = 0.002, I^2^ = 47%). Denosumab was also correlated with significantly higher overall survival rates at 18 months (99.2%) and 24 months (99.2%) compared to radiotherapy (91.5%; 89.6%) and SAE (92.5%; 89.4%) (*p* = 0.019, I^2^ = 8%; *p* = 0.004, I^2^ = 23%). Mortality was higher in patients receiving SAE (20.9%) or radiotherapy (13.6%) compared to denosumab (0.8%) (*p* < 0.001), but deaths mostly occurred for unrelated diseases. Conclusions: Denosumab, radiotherapy, and SAE are safe and effective for inoperable SGCTs. Clinical and radiological outcomes are mostly comparable, but denosumab may provide superior tumor control.

## 1. Introduction

Spine giant cell tumors (SGCTs) account for approximately 3% of all giant cell tumors of the bones (GCTBs), with a higher incidence in female patients aged 20–40 years [1,2]. Recently defined as intermediate malignant bone tumors, they are characterized by a osteolytic and locally aggressive nature, and commonly metastasize to the lungs [3]. Surgical en-bloc resection and intralesional curettage remain the treatments of choice, but the high rates of recurrence and postoperative functional sequelae pose serious limitations [4,5]. In addition, some patients with significant tumor burden or poor clinical status may be not eligible for surgery [6,7].

Non-surgical therapies have been investigated for inoperable SGCTs, with the goal of reducing morbidity while achieving comparable functional outcomes [6]. Due to the high radiosensitivity of SGCTs, locoregional radiotherapy has shown promising results in determining long-term local tumor control with minimal adverse events [8,9]. Selective arterial embolization (SAE) also proved to be effective in relieving pain, stabilizing lesions, and improving survival by causing tumor shrinkage and/or ossification, owing to the high vascularity of SGCTs [10,11]. More recently, denosumab has been approved for treating unresectable SGCTs, following its inhibitory activity against osteoclast activation and bone destruction, which often leads to favorable clinical and radiological outcomes [12,13,14]. 

Despite the promising role of such nonoperative options, no treatment guidelines have been defined for inoperable SGCTs [5,7]. Each strategy carries some risks of complications, and few negative prognostic factors have been identified. Only a few studies directly compared such strategies, reporting contradictory results [15]. In this study, we systematically reviewed the literature to assess the differences in clinical outcomes, radiological responses, tumor control, and survival between denosumab, radiotherapy, and SAE for treating patients with inoperable SGCTs.

## 2. Materials and Methods

### 2.1. Literature Search

A systematic review was conducted using the Preferred Reporting Items for Systematic Reviews and Meta-Analyses (PRISMA) guidelines [16] and registered to PROSPERO (ID: 303155). PubMed, EMBASE, Scopus, Web-of-Science, and Cochrane were searched from database inception to 30 August 2021, operating the Boolean full-text search (giant cell tumor AND (sacral OR vertebral OR spine OR spinal) AND (radiotherapy OR embolization OR denosumab)). Obtained studies were exported to Mendeley, and duplicates deleted.

### 2.2. Study Selection

A priori inclusion and exclusion criteria were determined. Articles were included if they: (1) included ≥4 patients receiving denosumab or locoregional radiotherapy or SAE for histologically confirmed SGCTs, namely involving mobile spine and/or the sacrum, not eligible to undergo surgical resection; (2) reported data on treatment outcomes and follow-up times; (3) were written in English. Inoperable SGCTs have been included as defined by the authors, namely cases not eligible to undergo surgery owing to the poor functional status of affected patients and/or inaccessible surgical access for tumor removal. Studies were excluded if they: (1) were literature reviews, technical notes, cadaver studies or animal studies; (2) involved patients receiving denosumab, radiotherapy or SAE as neoadjuvant or adjuvant strategies in surgical planning; (3) lacked data on treatment outcomes. 

Two reviewers (K.B. and P.P.) independently screened all titles and abstracts and then assessed full texts of articles that met the inclusion criteria. Disagreements were settled by a third reviewer (A.S.H.). Eligible papers were included, and references were screened to identify additional pertinent studies.

### 2.3. Data Extraction

One reviewer (A.C.) extracted data from each article, then confirmed independently by two additional reviewers (P.P. and N.S.S.). Missing data were not reported by the authors. Data included: author, study design, sample size, patients’ age and gender, tumor location, primary vs recurrence, prior non-surgical treatments for recurrent tumors, presenting symptoms, treatment strategies (denosumab vs radiotherapy vs SAE), adverse events, post-treatment clinical and radiological responses, SGCT local recurrence, distant metastases, follow-up, progression-free survival (PFS), overall survival (OS), survival status. Post-treatment symptom improvement (i.e., pain relief) and radiological responses were evaluated at 6 months or at last available follow-up. Radiological responses were assessed based on post-treatment changes in SGCT volumes as reported by the authors, denoting positive responses in patients with reduction in tumor size and negative responses in patients with no change or an increase in tumor size [17]. 

### 2.4. Data Synthesis and Quality Assessment

The primary outcomes of interest were clinical outcomes, radiological responses, tumor control, and survival based on treatment strategies. For each article, two independent authors (P.P. and N.S.S.) appraised the level of evidence using the 2011 Oxford Centre For Evidence-Based Medicine guidelines, and the risk of bias by applying the Joanna Briggs Institute checklists for case series and clinical trials [18,19,20].

### 2.5. Statistical Analysis

Statistical analyses were performed using STATA 17.0 (StataCorp LLC, College Station, TX, USA), and two-tailed *p*-values < 0.05 were considered statistically significant. Continuous variables are presented as medians and ranges, and categorical variables as percentages. Chi-square contingency analyses and ANOVA test group comparisons were conducted to test differences between treatment groups in categorical and continuous variables, respectively. Indirect meta-analyses were performed for post-treatment symptom improvement, radiological responses, treatment-related severe complications, local recurrence, distant metastases, and OS rates at 6-12-18-24 months. Outcomes were reported with pooled proportions of events (effect size, ES), and confidence intervals (CI) were estimated with the Wilson score method, both presented in forest plots [21]. The Freeman–Tukey transformation was performed to include studies with 0 or 1 event rates and stabilize variance [22]. The DerSimonian and Laird approach for random-effect models was used to account for high between-study variability [23]. Heterogeneity was assessed with the Higgins I-square (I^2^) and considered significant for I^2^ > 75% [24]. Publication bias was evaluated with generated funnel plots, which were examined for any evident visual asymmetry [25].

## 3. Results

### 3.1. Study Selection

**Figure 1:**
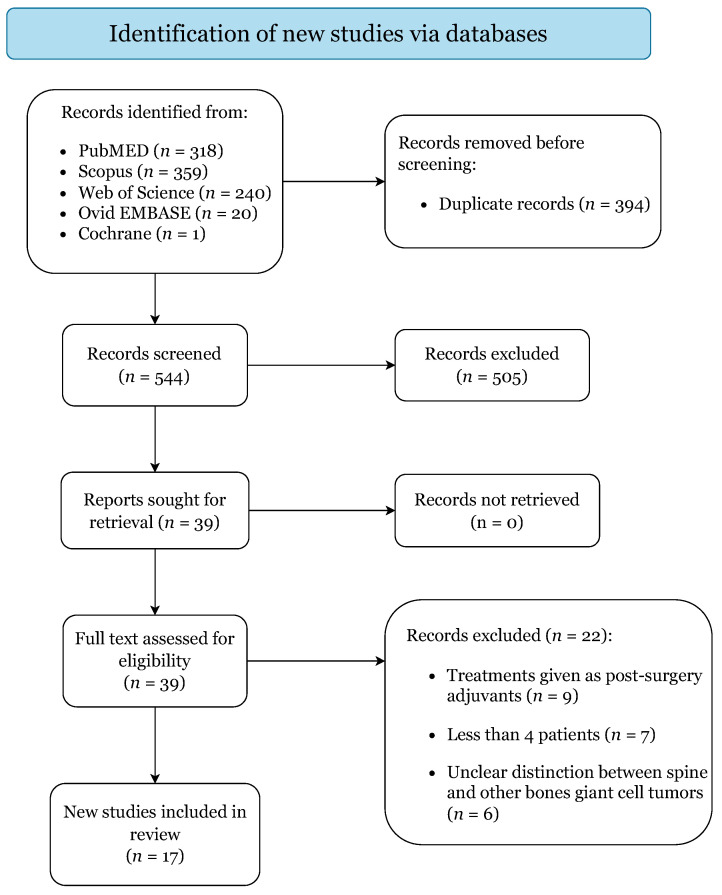
Preferred Reporting Items for Systematic Reviews and Meta-Analyses (PRISMA) 2020 Flow-Diagram.

Figure 1 illustrates the flow diagram of the study selection. The initial search yielded 938 studies (PubMed: 318, Scopus: 359, Web of Science: 240, Ovid-EMBASE: 20, Cochrane: 1), of which 16 case series and 1 open-label trial were included and categorized respectively as levels IV and IIb of evidence (Supplementary File S1) [9,11,12,14,26,27,28,29,30,31,32,33,34,35,36,37,38]. Quality assessment returned low risk of bias for all included studies (Supplementary File S2). Publication bias was excluded, as no evident visual asymmetry could be ascertained from the generated funnel plots (Supplementary File S3).

### 3.2. Demographics and Clinical Characteristics

A total of 230 patients diagnosed with single, biopsy-proven, and not previously resected SGCT were analyzed and grouped based on treatments: 128 (55.6%) received denosumab, 59 (25.7%) locoregional radiotherapy, and 43 (18.7%) SAE (Table 1). No significant differences in demographics and clinical features were found between the three groups, but SAE was only performed in patients with lumbar and sacral SGCTs (Table 2). Overall, median age was 34.5 years (range, 8–83) with a female prevalence (66.5%). Among the three groups, patients undergoing SAE were younger (median age 29.5 years), and patients receiving denosumab were older (median age 37.5 years). Most patients were treated for primary tumors (63.5%), while 84 patients (36.5%) had recurrent tumors previously managed nonoperatively with systemic chemotherapy. SCGTs were mostly located in the sacrum (64.8%), thoracic (14.3%), and cervical (11.3%) spine. All patients complained of locoregional pain related to tumor position, and 11 (8.9%) experienced motor impairments following spinal cord and/or nerve compression.

### 3.3. Management Strategies and Treatment Outcomes

In the denosumab cohort, patients completed monthly cycles of denosumab at the dosage of 120 mg for a median of 64.5 months (range, 5–102). In the radiotherapy cohort, all patients received photon locoregional radiation with a median maximal dose of 45Gy (range, 10.8–65) in a median of 21 fractions (range, 6–34). Schwartz et al. [28] reported one patient with a C1 tumor receiving a course of locoregional proton-therapy at 56 Gy following prior photon-therapy at 36Gy. In the SAE cohort, patients underwent selective embolization of the paraspinal arteries feeding the tumors with a median of 4 procedures (range, 1–10) per patient. All procedures were performed through a femoral access, mostly using steel coils and gelfoam particles as embolization means. Nakanishi et al. [36] described the use of superabsorbant polymer microspheres (SAP-MS), which are spherical permanent embolic materials, for SAE procedures in 4 patients with sacral SGCTs.

### 3.4. Treatment Outcomes and Survival

Median follow-up time was 69.3 months (range, 0.3–351), significantly longer in patients undergoing SAE (76.5 months; *p* < 0.001) (Table 2). At last follow-up, symptom improvement was described in 81.7% patients and positive radiological response in 62.2%, with no significant differences based on treatment (*p* = 0.219 and *p* = 0.105, respectively). A total of 29 patients (12.6%) experienced severe treatment-related adverse events, with no significant between-group differences (*p* = 0.313): 16 patients (12.5%) in the denosumab cohort, 5 (8.5%) in the radiotherapy cohort), and 8 (18.6%) in the SAE cohort. Denosumab mostly correlated with mandible osteoradionecrosis (7.8%) and malignancy transformation (3.1%), radiotherapy with skin/soft tissue disorders (5.1%) and severe sacral pain (3.4%), and SAE with focal neuropathy (14%) and spine instability (4.7%). Favorable rates of local and distant tumor control were reported among all treatment groups, with a median PFS of 12.3 months (range, 0.3–133): 43 patients (18.7%) had local recurrence and 8 (3.5%) had distant metastases. Overall, patients receiving denosumab had significantly lower rates of local recurrence (10.9%; *p* = 0.002) and distant metastases (0%; *p* = 0.021), but no differences in PFS were noted (*p* = 0.141). Median OS was 69.3 months (range, 0.3–351), significantly longer in patients undergoing SAE (*p* < 0.001). Survival rates were significantly superior in the denosumab cohort (99.2%) (*p* < 0.001), which was also correlated with significantly higher OS rates at 18 months (99.2%, *p* = 0.023) and 24 months (99.2%, *p* = 0.006). Deaths occurred in 18 patients (7.8%) for underlying conditions unrelated to their SGCTs diagnosis, mostly including old age, and cardiovascular and respiratory diseases.

### 3.5. Meta-Analysis: Comparison of Post-Treatment Outcomes Rates

**Figure 2:**
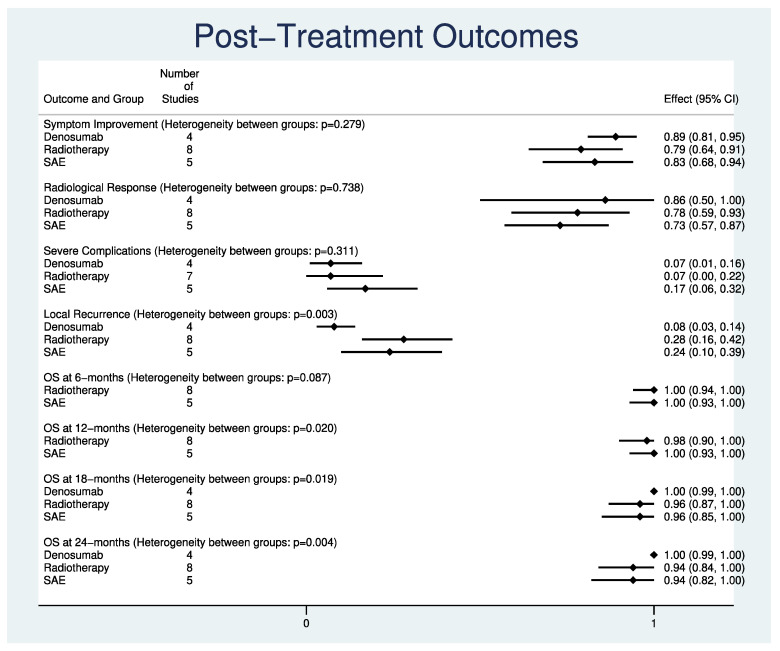
Combined forest plots for indirect comparisons between denosumab vs. radiotherapy vs. selective arterial embolization for giant cell tumor of the spine: symptom improvement; positive radiological response; severe complications; local recurrence; distant metastases; and overall survival at 6 months, 12 months, 18 months, and 24 months. Abbreviations: CI, confidence Interval; ES, Effect Size.

The results of all indirect comparisons between denosumab, radiotherapy, and SAE are summarized in Table 3 and displayed as combined forest plots in Figure 2 (individual forest plots can be found in Supplementary File S4). There were no significant differences in post-treatment symptom improvement (*p* = 0.279, I^2^ = 0%), positive radiological responses (*p* = 0.738, I^2^ = 56.8%), and severe complications (*p* = 0.311, I^2^ = 0%) between the three groups. Rates of local recurrence (*p* = 0.003; I^2^ = 32%) and distant metastases (*p* = 0.002; I^2^ = 47%) were significantly lower in patients receiving denosumab (ES:0.08, 95%CI:0.03–0.14 for local recurrence; ES:0.00, 95%CI:0.00–0.00 for distant metastases) as compared to radiotherapy (ES:0.28, 95%CI:0.16–0.42; ES: 0.06, 95%CI:0.00–0.21) and SAE (ES:0.24, 95%CI:0.10–0.39; ES:0.04; 95%CI:0.00–0.15).

While OS rates at 6 months (*p* = 0.087, I^2^ = 0%), were comparable between the three cohorts, OS rates at 12 months (*p* = 0.020, I^2^ = 0%), 18-months (*p* = 0.019, I^2^ = 8%), and 24 months (*p* = 0.004, I^2^ = 23.3%) were significantly higher in denosumab (ES:1.00, 95%CI:1.00–1.00; ES:1.00, 95%CI:0.99–1.00; ES:1.00, 95%CI:0.99–1.00) as compared to radiotherapy (ES:0.98, 95%CI:0.90–1.00; ES:0.96, 95%CI:0.87–1.00; ES:0.94, 95%CI:0.84–1.00) and SAE (ES:1.00, 95%CI:0.93–1.00; ES:0.96, 95%CI:0.85–1.00; ES:0.94, 95%CI:0.82–1.00).

## 4. Discussion

SGCTs are intermediate malignant bone tumors often responsible for debilitating axial pain and neurological impairments. While surgical resection represents the current standard treatment, less invasive strategies have been evaluated for inoperable cases [5,15]. This systematic review and meta-analysis sought to examine and compare denosumab, locoregional radiotherapy, and SAE in treating inoperable SGCTs, noting similar efficacy in providing favorable clinical and radiological outcomes with few treatment-related adverse events. However, patients receiving denosumab showed significantly longer rates of tumor control and survival.

GCTBs rarely involve the spine but may lead to important tumor burden due to their impact on patient functional status and risk of systemic metastases. In this review, we found that patients with SGCTs had comparable baseline characteristics regardless of the treatment group. Demographics were similar to the general population with GCTBs, mostly consisting of female patients in their second–fourth decades of life [5,7]. This implies that management strategies should be carefully planned to achieve symptom relief and disease control while preserving satisfactory long-term functional status in young patients with SGCTs, especially owing to their overall good prognosis. Most neoplasms involved the sacral region, likely due to their higher surgical risks compared to other spine SGCTs, which less commonly require non-surgical planning [15]. Furthermore, cervical and thoracic SGCTs have been reported to be even rarer entities, as we have also found among our included studies [39]. In our pooled patients, the presence of debilitating axial pain, often with concurrent neurological deficits, deemed necessary the pursuit of less invasive treatments aimed at relieving symptoms while minimizing the risks of surgical complications.

**Figure 3:**
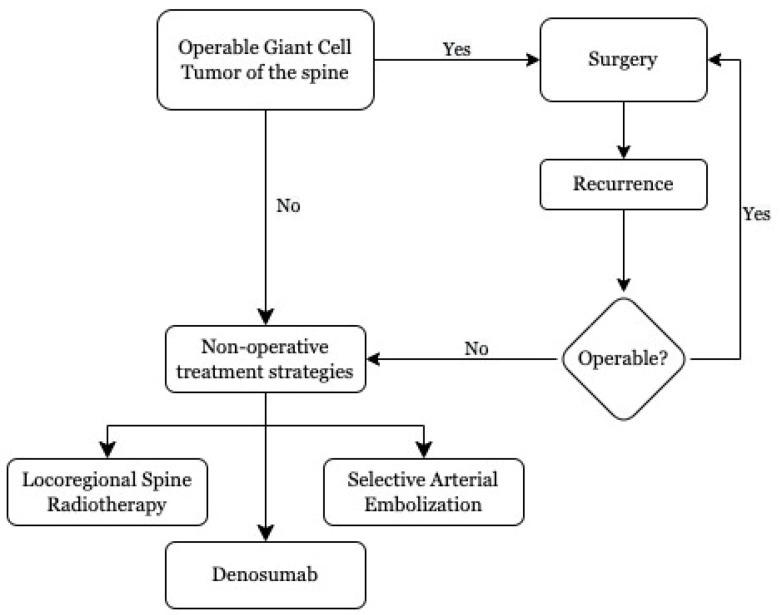
Treatment workflow in patients with spine giant cell tumors (SGCTs).

In line with other GCTBs, surgical resection and intralesional curettage constitute the treatments of choice in patients with SGCTs [5] (Figure 3). When feasible, total en-bloc resection is preferred because of its better local tumor control and lower recurrence rates [40,41,42,43]. However, aggressive procedures may result in severe permanent morbidity due to tumor proximity to critical paraspinal neurovascular structures and the risk of causing spine instability, thus necessitating spine fixation along with causing a major clinical burden in young patients. In particular, en-bloc resection of sacral SGCTs often requires sacrifice of the sacral nerve roots, which may lead to major disability with bladder and bowel dysfunction [44,45]. Intralesional curettage correlates with inferior risks of persistent neurological deficits but may still be associated with massive and difficult-to-control intraoperative bleeding and spine instability [4]. Hence, alternative nonoperative treatment options have been evaluated for inoperable SGCTs aimed at achieving favorable outcomes while minimizing the impact on patient neurological status [5,15].

Radiotherapy has been widely debated in SGCTs owing to their moderate radiosensitivity [34]. A recent systematic review reported a 100% response rate after therapeutic radiotherapy for inoperable SGCTs, but several studies showed variable rates of tumor control and radiation-induced complications, similarly to other GCTBs [5,8,9,30,32]. Megavoltage radiotherapy with a median dose of 45Gy was used in all our pooled cases, as it proved to be safer and more effective compared to orthovoltage radiotherapy for GCTBs [5,8]. Despite the lack of adequate data for SGCTs, newer intensity-modulated and stereotactic radiation techniques should also be evaluated for SGCTs for delivering targeted doses with lower complication risks [7].

Preoperative embolization of GCTBs may reduce the risk of intraoperative blood loss in intralesional curettage and tumor resection, thus improving surgical outcomes [10]. Contrarily, stand-alone SAE has been used for inoperable SGCTs to reduce tumor vascularity and achieve good size control with clinical improvement. Most studies included in this review reported planning serial embolization procedures with steel coils and gelfoam particles to obtain optimal management [33,35]. The use of microspheres, aimed at achieving optimal arterial occlusion in fewer SAE sessions, was initially described by Nakanishi et al. [36] in 4 hypervascular sacral SGCTs. Although the authors documented favorable outcomes in all patients, microspheres in SGCTs have not been further validated.

Denosumab was recently approved for the management of unresectable GCTBs due to its ability to inhibit osteoclastic-mediated bone destruction and reduce tumor blood supply [46,47]. Preoperatively, denosumab may downstage tumor size and reduce morbidity of surgical procedures; however, two systematic reviews found that neoadjuvant denosumab increases the risk of tumor recurrence after intralesional curettage, greatly limiting its usage [48,49]. In inoperable SGCTs, stand-alone denosumab is administered in 120 mg/monthly doses for prolonged periods, with a median time of 64.5 months found across our included studies. The frequent long-term courses of denosumab may be explained by the fact that higher recurrence rates have been noted after early stoppage of treatment, but prolonged exposure may also lead to increased risks of adverse events [5,15,17]. Hence, accurate planning for stand-alone denosumab in inoperable SGCTs is challenging and should be carefully evaluated weighting benefits and drawbacks in comparison to other treatment options. 

The primary treatment goals for GCTBs consist in providing long-term symptom relief with particular regard to pain, along with tumor control to maintain prolonged favorable functional status in affected patients [5]. In this review, we found that denosumab, locoregional radiotherapy, and SAE were associated with high rates of symptom improvement and pain relief in patients with inoperable SGCTs, similarly to previous reports on other inoperable GCTBs and/or resected SGCTs [8,10,17]. Of interest, no significant difference was found between the three strategies (*p* = 0.279), with modest clinical advantages correlated to denosumab (85.2%) over radiotherapy (74.6%) and SAE (81.4%), likely owing to its direct action against osteoclastic bone destruction, which is the primary culprit responsible for axial pain [17,47,50]. For post-treatment radiologic tumor changes, all three strategies had good and comparable (*p* = 0.738) rates of reduction in tumor volume, probably accountable for the clinical improvement noted in most cases. We presume that the moderately lower rates of tumor size decrease found in patients treated with denosumab (56.3%), as compared to radiotherapy (67.8%) and SAE (72%), were possibly attributed to the somewhat variable radiologic outcomes observed across all included studies. Indeed, post-treatment SGCTs radiologic outcomes comprised changes in volumes of bone and soft-tissue components, arrest in bone lysis, and central sclerosis formation [12,14,37,38]. As described in histopathology reports, denosumab efficiently destroys reactive osteoclastic cells in SGCTs but has a limited effect in eliminating neoplastic stromal cells, only slowing their proliferation rates, which may further explain its lower rates of tumor volume reduction [51,52]. 

We found that patients treated with denosumab had significantly lower rates of local recurrence (10.9%; *p* = 0.003) and distant metastases (0%; *p* = 0.002) in comparison to radiotherapy (30.5%; 8.5%) and SAE (25.6%; 7%). We hypothesize this to be related to the different duration-related mechanisms-of-action of each therapy: while the prolonged use of denosumab directly eliminates RANK-positive reactive giant cells and makes stromal tumor cells quiescent during its whole therapeutic course, radiotherapy and SAE, performed in single or in a limited number of procedures, indirectly and temporarily reduce tumor nutrition and cellular activity, likely capable of regrowing and/or metastasizing [8,10,47]. The few post-denosumab recurrences found across our included studies occurred in patients with early cessation of treatment, further supporting previous findings showing superior local control rates with prolonged courses of denosumab [12,14,38,53]. Our pooled local recurrence rates were also inferior to the ones reported in SGCTs treated with neoadjuvant denosumab and intralesional curettage (43%), and similar to those in patients receiving stand-alone resection or curettage (10–25%) [5,49]. Although some cases of distant metastases occurred after radiotherapy or SAE, rates were comparable to those after GCTB resection (1–9%), probably suggesting the underlying malignant nature of some tumors and/or their inevitable disease evolution [5]. Hence, all three strategies may be considered as valuable alternative to surgery in inoperable SGCTs, showing similar efficacy for achieving optimal treatment goals. 

Patients with GCTBs, including SGCTs, have a good prognosis overall, with cancer-related mortality mostly occurring in the few cases of tumor progression, malignant transformation or metastasis [5,15]. Consequently, the role of available treatment strategies is to preserve long-term functional status in affected patients and minimize the risk of treatment-related, life-threatening events [7,39]. In our pooled data, the median OS was significantly longer in the SAE cohort (76.5 months) as compared to the radiotherapy (69.2 months) and denosumab (66.9 months) cohorts (*p* < 0.001), likely due to the statistically different follow-up time between the three groups (*p* < 0.001). However, survival times were comparable to those observed in surgically treated SGCTs, further highlighting the clinical benefits of all three nonoperative strategies. Although survival rates were significantly superior at 12 months (*p* = 0.020), 18 months (*p* = 0.019), and 24 months (*p* = 0.006) in the denosumab cohort, we note that all deaths occurred for non-oncological and unrelated conditions, comprising older age and medical comorbidities [11,26,27,34]. In addition, years of publication were largely different among the three treatments, more recent for denosumab studies, which may have contributed to such survival differences following the important advances in the management of oncological patients introduced during the past decade [54,55]. 

The major concerns in treating patients with SGCTs include the risks of treatment-related adverse events. In this review, we found that all three strategies showed safe therapeutic profiles and low rates of severe complications, with no significant between-group differences (*p* = 0.311). In addition, our pooled rates were inferior to those reported in previous surgical series (up to 35% for sacral SGCTs) [4,5,7]. We note that radiotherapy and SAE complications were likely subjected to publication bias. Indeed, recent delivery techniques in spine oncology significantly reduced the risks of radiation-induced skin disorders and sacral pain [56]. Similarly, newer spine embolization approaches notably decreased the occurrence of embolization-related post-surgical access neuropathies and spine instability [57]. Regarding denosumab, our pooled complications were similar to the general adverse events reported for other medical uses of denosumab, mostly consisting of jaw osteoradionecrosis occurring after prolonged treatment courses in patients with concurrent oral/dental comorbidities or surgery [14,17,47]. Finally, 4 cases of SGCTs malignant transformation were noted during denosumab therapy, but sampling error at the time of initial biopsy may have been responsible [14]. Hence, although some recent studies have questioned the clinical benefit of denosumab in GCTBs [48], especially in regard to its related adverse events after tumor curettage, in this review we found that denosumab is a safe and effective primary treatment in patients with SGCTs, showing favorable clinical-radiological outcomes and low complication rates. We ascribe such differences to potential underlying histomolecular characteristics unique to SGCTs as compared to GCTBs, which deserve further evaluation to optimize disease-specific treatment strategies and protocols.

### Limitations

Our study has limitations. Except for one open-label single-arm trial, all included studies were retrospective with class IV evidence, prone to selection and recall biases. Numbers of included patients for each cohort were different, likely limiting the power of our meta-analysis. Possible clinical confounders could not be investigated due to the limited data on medical comorbidities and lack of standardized evaluation of baseline and post-treatment patient performance status. The difference in time period of study publication and follow-up intervals between the three strategies may have introduced some confounding variables into our survival analysis. Finally, we note that our strict study selection criteria excluded all studies reporting the use of these nonoperative treatment strategies as second-line and third-line options in patients with post-surgical relapsing SGCTs. However, our inclusion criteria were set a priori to prevent the introduction of additional confounding variables into our meta-analysis, which may have been related to the difficulty in differentiating the clinical outcomes based on each individual treatment option and to the heterogeneity in clinical responses between primary and relapsing SGCTs.

## 5. Conclusions

This meta-analysis compared the roles of denosumab, radiotherapy, and SAE for inoperable SGCTs. All strategies showed safe toxicity profiles and similar efficacy in providing pain relief and favorable radiological responses, comparable to those observed in surgical series. Denosumab resulted in significantly longer tumor control and survival, but prolonged courses and concurrent medical comorbidities should be carefully considered to minimize the risk of adverse events. Future studies should analyze and compare the roles of denosumab, radiotherapy, and SAEs also as second-line and third-line options in patients with post-surgery relapsing SGCTs.

## Figures and Tables

**Table 1:** Summary of clinical features, management, and treatment outcomes of all pooled patients.

Characteristics	Value
Cohort size (no.)	230
Demographics	
Age (years), median (range)	34.5, 8–83
Gender (female)	153 (66.5%)
Type Tumor	
Primary	146 (63.5%)
Recurrent	84 (36.5%)
Tumor Location	
Cervical	26 (11.3%)
Cervical-Thoracic	1 (0.4%)
Thoracic	33 (14.3%)
Thoraco-Lumbar	2 (0.9%)
Lumbar	16 (7%)
Lumbo-Sacral	3 (1.3%)
Sacral	149 (64.8%)
Treatment Characteristics	
Denosumab	128 (55.7%)
Dose	120 mg/monthly
Duration of treatment (months), median (range)	64 (0.5–102)
Radiotherapy	59 (25.7%)
Photon therapy	52 (88.1%)
Proton therapy	7 (11.9%)
Dose (Gy), median (range)	45 (10.8–65)
Selective Arterial Embolization	43 (18.7%)
Gelfoam + Steel Coil	39 (90.7%)
Superabsorbant polymer microsphere (SAP-MS)	4 (9.3%)
Number of procedures per-patient, median (range)	4 (1–10)
Severe Complications	29 (12.6%)
Osteonecrosis mandible	10 (4.3%)
Neuropathy	6 (2.6%)
Malignant transformation	4 (1.7%)
Skin/soft tissue disorders	3 (1.3%)
Severe sacral pain	2 (0.9%)
Spine instability	2 (0.9%)
Femur fracture	1 (0.4%)
Severe hypercalcemia	1 (0.4%)
Clinical-Radiological outcomes	
Symptom improvement	188 (81.7%)
Positive radiological response	143 (62.2%)
Tumor Control	
Local recurrence	43 (18.7%)
Distal metastases	8 (3.5%)
Lung	6 (2.6%)
Bone	1 (0.4%)
Soft tissue	1 (0.4%)
Survival outcomes	
Follow-up (months), median (range)	69.3 (0.3–351)
Progression-free survival (months), median (range)	12.3 (0.3–133)
Overall survival (months), median (range)	69.3 (0.3–351)
At 6-months	99.1%
At 12-months	98.2%
At 18-months	95.9%
At 24-months	94.9%
Status	
Alive	212 (92.2%)
Dead	18 (7.8%)

**Table 2:** Summary and comparisons of all pooled patients grouped by treatment cohort.

Characteristics	Denosumab	Radiotherapy	SAE	*p*-Value
Cohort size (no.)	128	59	43	
Demographics				
Age (years), median (range)	37.5 (12–83)	31.5 (8–77)	29.5 (15–68)	0.082 *
Gender (female)	83 (64.8%)	37 (62.7%)	33 (76.7%)	0.277 ^
Type of Tumor				0.220 ^
Primary	77 (60.1%)	43 (72.9%)	26 (60.5%)	
Recurrent	51 (39.9%)	16 (27.1%)	17 (39.5%)	
Tumor Location				0.078 ^
Cervical	17 (13.3%)	9 (15.2%)	0 (0%)	
Cervical-Thoracic	0 (0%)	1 (1.7%)	0 (0%)	
Thoracic	19 (14.8%)	14 (28.6%)	0 (0%)	
Thoraco-Lumbar	2 (1.6%)	0 (0%)	0 (0%)	
Lumbar	10 (7.8%)	5 (8.5%)	1 (2.3%)	
Lumbo-Sacral	0 (0%)	1 (1.7%)	2 (4.7%)	
Sacral	80 (62.5%)	29 (49.2%)	40 (93%)	
Severe Complications				
Total	16 (12.5%)	5 (8.5%)	8 (18.6%)	0.313 ^
Osteonecrosis mandible	10 (7.8%)	0 (0%)	0 (0%)	
Neuropathy	0 (0%)	0 (0%)	6 (14%)	
Malignancy transformation	4 (3.1%)	0 (0%)	0 (0%)	
Skin/soft tissue disorders	0 (0%)	3 (5.1%)	0 (0%)	
Severe sacral pain	0 (0%)	2 (3.4%)	0 (0%)	
Spine instability	0 (0%)	0 (0%)	2 (4.7%)	
Femur fracture	1 (0.8%)	0 (0%)	0 (0%)	
Severe hypercalcemia	1 (0.8%)	0 (0%)	0 (0%)	
Clinical-Radiological Outcomes				
Symptom improvement	109 (85.2%)	44 (74.6%)	35 (81.4%)	0.219 ^
Radiological tumor response	72 (56.3%)	40 (67.8%)	31 (72%)	0.105 ^
Tumor Control				
Local recurrence	14 (10.9%)	18 (30.5%)	11 (25.6%)	**0.002 ^**
Distant metastases	0 (0%)	5 (8.5%)	3 (7%)	**0.021 ^**
Survival Outcomes				
Follow-up (months)	66.9 (4–102)	69.2 (1–351)	76.5 (0.3–277)	<0.001 *
PFS (months), median (range)	12 (2–15)	11.5 (5–72)	14 (0.3–133)	0.141 *
OS (months), median (range)	66.9 (4–102	69.2 (1–351)	76.5 (0.3–277)	<0.001 *
At 6-months	100%	98.3%	97.6%	0.706 ^
At 12-months	100%	94.9%	97.6%	0.186 ^
At 18-months	99.2%	91.5%	92.5%	**0.023 ^**
At 24-months	99.2%	89.6%	89.4%	**0.006 ^**
Status				**<0.001 ^**
Alive	127 (99.2%)	51 (86.4%)	34 (79.1%)	
Dead	1 (0.8%)	8 (13.6%)	9 (20.9%)	

* ANOVA test; ^ Chi-square test. *p* < 0.05 set for statistical significance. Bold for significant differences. Abbreviations: N/A, Not applicable; OS, Overall survival; PFS, Progression free survival; SAE, Selective arterial embolization.

**Table 3:** Summary of indirect comparisons between denosumab, radiotherapy, and selective arterial embolization.

Post-Treatment Outcomes Rates	Denosumab ES (95% CI)	Radiotherapy ES (95% CI)	SAE ES (95% CI)	*p*-Value * (I^2^%)
Clinical				
Symptom Improvement	0.89 (0.81–0.95)	0.79 (0.64–0.91)	0.83 (0.68–0.94)	0.279 (0%)
Positive Radiological Response	0.86 (0.50–1.00)	0.78 (0.59–0.63)	0.73 (0.57–0.87)	0.738 (56.8%)
Severe Complications	0.07 (0.01–0.16)	0.07 (0.00–0.22)	0.17 (0.06–0.32)	0.311 (0%)
Tumor Control				
Local Recurrence	77 (60.1%)	43 (72.9%)	26 (60.5%)	**0.003** (32%)
Distant Metastases	51 (39.9%)	16 (27.1%)	17 (39.5%)	**0.002** (47%)
Overall Survival				
6 Months	1.00 (1.00–1.00)	1.00 (0.94–1.00)	1.00 (0.93–1.00)	0.087 (0%)
12 Months	1.00 (1.00–1.00)	0.98 (0.90–1.00)	1.00 (0.93–1.00)	**0.020** (0%)
18 Months	1.00 (0.99–1.00)	0.96 (0.87–1.00)	0.96 (0.85–1.00)	**0.019** (8%)
24 Months	1.00 (0.99–1.00)	0.94 (0.84–1.00)	0.94 (0.82–1.00)	**0.004** (23.3%)

* Indirect meta-analysis with random-effect modeling. *p* < 0.05 was considered statistically significant for all tests; Heterogeneity I^2^ > 75% was considered significant. Bold for significant results. All survival data are reported from the time of starting treatment. Abbreviations: 95% CI, 95% confidence interval; ES, effect size; SAE, selective arterial embolization.

## Supplementary Materials

The following are available online at https://www.mdpi.com/article/10.3390/cancers14040937/s1, Supplementary File S1: Overview of all included studies, Supplementary File S2: Risk of bias assessments for all included studies, Supplementary File S3: Funnel plots, Supplementary File S4: Individual forest plots.

## References

[1] Mendenhall W.M., Zlotecki R.A., Scarborough M.T., Gibbs C.P., Mendenhall N.P. (2006). Giant Cell Tumor of Bone. Am. J. Clin. Oncol..

[2] Orguc S., Arkun R. (2014). Primary Tumors of the Spine. Semin. Musculoskelet. Radiol..

[3] WHO Classification of Tumours Editorial Board (2020). Soft Tissue and Bone Tumours.

[4] Luksanapruksa P., Buchowski J.M., Singhatanadgige W., Bumpass D.B. (2016). Systematic Review and Meta-analysis of En Bloc Vertebrectomy Compared with Intralesional Resection for Giant Cell Tumors of the Mobile Spine. Glob. Spine J..

[5] Tsukamoto S., Mavrogenis A.F., Kido A., Errani C. (2021). Current Concepts in the Treatment of Giant Cell Tumors of Bone. Cancers.

[6] Charest-Morin R., Boriani S., Fisher C.G., Patel S.R., Kawahara N., Mendel E., Bettegowda C., Rhines L.D. (2016). Benign Tumors of the Spine. Spine (Phila. Pa. 1976)..

[7] Luksanapruksa P., Buchowski J.M., Singhatanadgige W., Rose P.C., Bumpass D.B. (2016). Management of spinal giant cell tumors. Spine J..

[8] Ma Y., Xu W., Yin H., Huang Q., Liu T., Yang X., Wei H., Xiao J. (2015). Therapeutic radiotherapy for giant cell tumor of the spine: A systemic review. Eur. Spine J..

[9] Nair M.K., Jyothirmayi R. (1999). Radiation therapy in the treatment of giant cell tumor of bone. Int. J. Radiat. Oncol. Biol. Phys..

[10] He S., Xu W., Sun Z., Liu W., Liu Y., Wei H., Xiao J. (2017). Selective Arterial Embolization for the Treatment of Sacral and Pelvic Giant Cell Tumor: A Systematic Review. Orthop. Surg..

[11] Lin P.P., Guzel V.B., Moura M.F., Wallace S., Benjamin R.S., Weber K.L., Morello F.A., Gokaslan Z.L., Yasko A.W. (2002). Long-term follow-up of patients with giant cell tumor of the sacrum treated with selective arterial embolization. Cancer.

[12] Boriani S., Cecchinato R., Cuzzocrea F., Bandiera S., Gambarotti M., Gasbarrini A. (2020). Denosumab in the treatment of giant cell tumor of the spine. Preliminary report, review of the literature and protocol proposal. Eur. Spine J..

[13] Xará-Leite F., Coutinho L., Fleming C., Magalhães M., Oliveira V., Rodrigues-Pinto R., Cardoso P. (2020). Can Denosumab cure giant cell tumors of the spine? A case report and literature review. Eur. J. Orthop. Surg. Traumatol..

[14] Bukata S.V., Blay J.-Y., Rutkowski P., Skubitz K., Henshaw R., Seeger L., Dai T., Jandial D., Chawla S. (2021). Denosumab Treatment for Giant Cell Tumor of the Spine Including the Sacrum. Spine (Phila. Pa. 1976).

[15] Puri A., Gupta S.M., Gulia A., Shetty N., Laskar S. (2020). Giant cell tumors of the sacrum: Is non-operative treatment effective?. Eur. Spine J..

[16] Page M.J., McKenzie J.E., Bossuyt P.M., Boutron I., Hoffmann T.C., Mulrow C.D., Shamseer L., Tetzlaff J.M., Akl E.A., Brennan S.E. (2021). The PRISMA 2020 statement: An updated guideline for reporting systematic reviews. BMJ.

[17] Luengo-Alonso G., Mellado-Romero M., Shemesh S., Ramos-Pascua L., Pretell-Mazzini J. (2019). Denosumab treatment for giant-cell tumor of bone: A systematic review of the literature. Arch. Orthop. Trauma Surg..

[18] Howick J., Chalmers I., Glasziou P., Greenhalgh T., Heneghan C., Liberati A., Moschetti I., Phillips B., Thornton H. Explanation of the 2011 Oxford Centre for Evidence-Based Medicine (OCEBM) Levels of Evidence (Background Document). https://www.cebm.ox.ac.uk/resources/levels-of-evidence/ocebm-levels-of-evidence.

[19] Briggs J. Institute Checklist for Case Series. https://jbi.global/sites/default/files/2019-05/JBI_Critical_Appraisal-Checklist_for_Case_Series2017_0.pdf.

[20] Briggs J. Institute Checklist for Randomized Controlled Trials. https://jbi.global/sites/default/files/2019-05/JBI_RCTs_Appraisal_tool2017_0.pdf.

[21] Wilson E.B. (1927). Probable Inference, the Law of Succession, and Statistical Inference. J. Am. Stat. Assoc..

[22] Freeman M.F., Tukey J.W. (1950). Transformations Related to the Angular and the Square Root. Ann. Math. Stat..

[23] DerSimonian R., Laird N. (1986). Meta-analysis in clinical trials. Control. Clin. Trials.

[24] Higgins J.P.T. (2003). Measuring inconsistency in meta-analyses. BMJ.

[25] Sterne J.A., Egger M. (2001). Funnel plots for detecting bias in meta-analysis: Guidelines on choice of axis. J. Clin. Epidemiol..

[26] Chuang V., Soo C., Wallace S., Benjamin R. (1981). Arterial occlusion: Management of giant cell tumor and aneurysmal bone cyst. Am. J. Roentgenol..

[27] Seider M.J., Rich T.A., Ayala A.G., Murray J.A. (1986). Giant cell tumors of bone: Treatment with radiation therapy. Radiology.

[28] Schwartz L.H., Okunieff P.G., Rosenberg A., Suit H.D. (1989). Radiation therapy in the treatment of difficult giant cell tumors. Int. J. Radiat. Oncol..

[29] Bennett C.J., Marcus R.B., Million R.R., Eenneking W.F. (1993). Radiation therapy for giant cell tumor of bone. Int. J. Radiat. Oncol..

[30] Turcotte R.E., Sim F.H., Unni K.K. (1993). Giant cell tumor of the sacrum. Clin. Orthop. Relat. Res..

[31] Hug E.B., Fitzek M.M., Liebsch N.J., Munzenrider J.E. (1995). Locally challenging osteo- and chondrogenic tumors of the axial skeleton: Results of combined proton and photon radiation therapy using three-dimensional treatment planning. Int. J. Radiat. Oncol..

[32] Malone S., O’Sullivan B., Catton C., Bell R., Fornasier V., Davis A. (1995). Long-term follow-up of efficacy and safety of megavoltage radiotherapy in high-risk giant cell tumors of bone. Int. J. Radiat. Oncol..

[33] Lackman R.D., Khoury L.D., Esmail A., Donthineni-Rao R. (2002). The treatment of sacral giant-cell tumours by serial arterial embolisation. J. Bone Jt. Surg. Br..

[34] Caudell J.J., Ballo M.T., Zagars G.K., Lewis V.O., Weber K.L., Lin P.P., Marco R.A., El-Naggar A.K., Benjamin R.S., Yasko A.W. (2003). Radiotherapy in the management of giant cell tumor of bone. Int. J. Radiat. Oncol..

[35] Hosalkar H.S., Jones K.J., King J.J., Lackman R.D. (2007). Serial Arterial Embolization for Large Sacral Giant-Cell Tumors. Spine (Phila. Pa. 1976)..

[36] Nakanishi K., Osuga K., Hori S., Hamada K., Hashimoto N., Araki N., Yoshikawa H., Tomiyama N. (2013). Transarterial embolization (TAE) of sacral giant cell Tumor (GCT) using spherical parmanent embolic material superabsorbant polymer microsphere (SAP-MS). Springerplus.

[37] Goldschlager T., Dea N., Boyd M., Reynolds J., Patel S., Rhines L.D., Mendel E., Pacheco M., Ramos E., Mattei T.A. (2015). Giant cell tumors of the spine: Has denosumab changed the treatment paradigm?. J. Neurosurg. Spine.

[38] Sambri A., Medellin M.R., Errani C., Campanacci L., Fujiwara T., Donati D., Parry M., Grimer R. (2020). Denosumab in giant cell tumour of bone in the pelvis and sacrum: Long-term therapy or bone resection?. J. Orthop. Sci..

[39] Müther M., Schwake M., Suero Molina E., Schroeteler J., Stummer W., Klingenhöfer M., Ewelt C. (2021). Multiprofessional Management of Giant Cell Tumors in the Cervical Spine: A Systematic Review. World Neurosurg..

[40] Boriani S., Bandiera S., Casadei R., Boriani L., Donthineni R., Gasbarrini A., Pignotti E., Biagini R., Schwab J.H. (2012). Giant Cell Tumor of the Mobile Spine. Spine (Phila. Pa. 1976).

[41] Yokogawa N., Murakami H., Demura S., Kato S., Yoshioka K., Shimizu T., Oku N., Kitagawa R., Tsuchiya H. (2018). Total spondylectomy for Enneking stage III giant cell tumor of the mobile spine. Eur. Spine J..

[42] Passanisi M., Scalia G., Palmisciano P., Franceschini D., Crea A., Capone C., Tranchina M.G., Nicoletti G.F., Cicero S., Umana G.E. (2021). Difficulty differentiating between a posterior extradural lumbar tumor versus sequestered disc even with gadolinum-enhanced MRI. Surg. Neurol. Int..

[43] Sagoo N.S., Haider A.S., Palmisciano P., Vannabouathong C., Gonzalez R., Chen A.L., Lokesh N., Sharma N., Larsen K., Singh R. (2021). Coccygectomy for refractory coccygodynia: A systematic review and meta-analysis. Eur. Spine J..

[44] Li D., Guo W., Tang X., Ji T., Zhang Y. (2011). Surgical classification of different types of en bloc resection for primary malignant sacral tumors. Eur. Spine J..

[45] Umana G.E., Scalia G., Palmisciano P., Passanisi M., Pompili G., Amico P., Ippolito M., Sabini M.G., Cicero S., Perrotta R. (2021). Spontaneous sacral fracture with associated acrometastasis of the hand. Surg. Neurol. Int..

[46] Lewin J., Thomas D.M. (2013). Denosumab: A new treatment option for giant cell tumor of bone. Drugs Today.

[47] Niu X., Yang Y., Wong K.C., Huang Z., Ding Y., Zhang W. (2019). Giant cell tumour of the bone treated with denosumab: How has the blood supply and oncological prognosis of the tumour changed?. J. Orthop. Transl..

[48] Tsukamoto S., Tanaka Y., Mavrogenis A.F., Kido A., Kawaguchi M., Errani C. (2020). Is Treatment with Denosumab Associated with Local Recurrence in Patients with Giant Cell Tumor of Bone Treated with Curettage? A Systematic Review. Clin. Orthop. Relat. Res..

[49] Zhao Y., Cai Z., Tang X., Du Z., Yang Y., Guo W. (2021). Preoperative Denosumab may increase the Risk of Local Recurrence of Giant-cell Tumor of Bone Treated with Curettage: A Systematic Review and Meta-analysis. J. Cancer.

[50] Kane C.M., Hoskin P., Bennett M.I. (2015). Cancer induced bone pain. BMJ.

[51] Mak I.W.Y., Evaniew N., Popovic S., Tozer R., Ghert M. (2014). A Translational Study of the Neoplastic Cells of Giant Cell Tumor of Bone Following Neoadjuvant Denosumab. J. Bone Jt. Surg..

[52] Branstetter D.G., Nelson S.D., Manivel J.C., Blay J.-Y., Chawla S., Thomas D.M., Jun S., Jacobs I. (2012). Denosumab Induces Tumor Reduction and Bone Formation in Patients with Giant-Cell Tumor of Bone. Clin. Cancer Res..

[53] Matcuk G.R., Patel D.B., Schein A.J., White E.A., Menendez L.R. (2015). Giant cell tumor: Rapid recurrence after cessation of long-term denosumab therapy. Skelet. Radiol..

[54] Kalia M. (2013). Personalized oncology: Recent advances and future challenges. Metabolism.

[55] Palmisciano P., Haider A.S., Sabahi M., Nwagwu C.D., Bin Alamer O., Scalia G., Umana G.E., Cohen-Gadol A.A., El Ahmadieh T.Y., Yu K. (2021). Primary Skull Base Chondrosarcomas: A Systematic Review. Cancers.

[56] Cox B.W., Spratt D.E., Lovelock M., Bilsky M.H., Lis E., Ryu S., Sheehan J., Gerszten P.C., Chang E., Gibbs I. (2012). International Spine Radiosurgery Consortium Consensus Guidelines for Target Volume Definition in Spinal Stereotactic Radiosurgery. Int. J. Radiat. Oncol..

[57] Luksanapruksa P., Buchowski J.M., Tongsai S., Singhatanadgige W., Jennings J.W. (2018). Systematic review and meta-analysis of effectiveness of preoperative embolization in surgery for metastatic spine disease. J. Neurointerv. Surg..

