# Supplementary material for: Evaluating the Optimal Management of Inoperable Giant Cell Tumors of the Spine: A Systematic Review and Meta-Analysis
Source: Cancers (Basel). 2022 Feb 14;14(4):937. doi: 10.3390/cancers14040937 (PMC8870612; doi:10.3390/cancers14040937)
Supplement: Supplementary file 1 [file cancers-14-00937-s001.zip › cancers-1574019 Supplementary File.pdf]

Supplementary File S1. Overview of all included studies.

	Authors – Year	Study Design – Level of Evidence	Type of Therapy	Patients No./ Female	Age years median (range)	Location No. patients (percentage)	Primary or Recurrent No. patients (percentage)	Adverse Events No. patients (percentage)	Symptom Improvement No. patients (percentage)	Radiological Response No. patients (percentage)	Recurrence of SGCT No. patients (percentage)	PFS months median (range)	OS months median (range)	Survival Status No. patients (percentage)
1	**Chang et al. – 1981**[24]	Case series – IV	SAE	7 / 6 (85.7%)	33 (13 – 57)	4 Sacral (57.1%), 2 L-S (28.6%), 1 Lumbar (14.3%)	7 Recurrent (100%)	2 N (28.6%), 1 MI (14.3%), 1 SI (14.3%)	6 (85.7%)	6 PR (85.7%), 1 PD (14.3%)	1 (14.3%)	0.3	14.0 (0.3 – 55.0)	6 Alive (85.7%)
2	**Seider et al. – 1986**[14]	Case series – IV	RT	7 / 7 (100%)	29 (12 – 49)	5 Sacral (71.4%), 1 Lumbar (14.3%), 1 L-S (14.3%)	6 Recurrent (85.7%), 1 Primary (14.3%)	1 DWH (14.3%), 1 SF (14.3%), 1 SN (14.3%)	4 (57.1%)	4 PR (57.1%), 3 PD (42.9%)	3 (42.9%)	12.0 (12.0 – 72.0)	99.0 (23.0 – 351.0)	4 Alive (57.1%)
3	**Schwartz et al. – 1989**[32]	Case series – IV	RT	4 / 2 (50%)	45 (26 – 56)	2 Thoracic (50%), 1 Cervical (25%), 1 Sacral (25%)	4 Primary (100%)	0 (0%)	4 (100%)	4 PR (100%)	1 (25%)	5.0	24.0 (18.0 – 72.0)	4 Alive (66.7%)
4	**Bennett et al. – 1993**[25]	Case series – IV	RT	6 / 2 (33.3%)	40 (17 – 43)	3 Sacral (50%), 2 Cervical (33.3%), 1 Thoracic (16.7%)	6 Primary (100%)	2 SSP (33.3%)	5 (83.3%)	5 PR (83.3%), 1 PD (16.7%)	1 (16.7%)	12.0	57.5 (32.0 – 160.0)	6 Alive (1007%)
5	**Turcotte et al. – 1993**[12]	Case series – IV	RT	5 / 4 (80%)	29 (15 – 77)	5 Sacral (100%)	5 Primary (100%)	0 (0%)	4 (80%)	4 PR (80%), 1 PD (20%)	1 (20%)	12.0	14.0 (12.0 – 160.0)	5 Alive (100%)
6	**Hug et al. – 1995**[26]	Case series – IV	RT	6 / 3 (50%)	25 (8 – 76)	3 Cervical (50%), 3 Sacral (50%)	6 Primary (100%)	0 (0%)	4 (66.7%)	5 PR (83.3%), 1 PD (16.7%)	2 (33.3%)	12.0	38.0 (6.0 – 136.0)	6 Alive (100%)
7	**Malone et al – 1995**[33]	Case series – IV	RT	6 / 4 (66.7%)	29 (13 – 52)	3 Thoracic (50%), 2 Sacral (33.3%), 1 Lumbar (16.7%)	6 Primary (100%)	0 (0%)	6 (100%)	5 PR (83.3%), 1 PD (16.7%)	0 (0%)	N/A	100.0 (34.0 – 298.0)	6 Alive (100%)
8	**Nair et al. – 1999**[15]	Case series – IV	RT	5 / 2 (40%)	27 (18 – 65)	2 Thoracic (40%), 2 Sacral (40%), 1 Cervical (20%)	5 Primary (100%)	0 (0%)	4 (80%)	4 PR (80%), 1 PD (20%)	1 (20%)	18.0	26.0 (6.0 – 156.0)	3 Alive (40%)
9	**Lackman et al. – 2002**[27]	Case series – IV	SAE	5 / 4 (80%)	21 (19 – 42)	5 Sacral (100%)	5 Primary (100%)	1 SpI (20%)	4 (80%)	4 PR (80%), 1 PD (20%)	1 (20%)	6.0	60.0 (18.0 – 202.0)	4 Alive (80%)
10	**Lin et al. – 2002**[13]	Case series – IV	SAE	18 / 13 (72.2%)	30 (15 – 59)	18 Sacral (100%)	10 Primary (55.5%), 8 Recurrent	2 MI (11.1%), 1 SI (5.6%)	14 (77.8%)	11 PR (61.1%), 7 PD (38.9%)	7 (38.9%)	16.0 (5.0 – 133.0)	96.5 (12.0 – 277.0)	13 Alive (72.2%)

							(44.5%)							
11	**Caudell et al. – 2003**[28]	Case series – IV	RT	20 / 13 (65%)	31.5 (13 – 63)	8 Sacral (40%), 6 Thoracic (30%), 3 Lumbar (15%), 2 Cervical (10%), 1 C-T (5%)	10 Primary (50%), 10 Recurrent (50%)	0 (0%)	12 60%)	11 PD (55%), 9 PR (45%)	9 (45%)	N/A	96.0 (1.0 – 348.0)	17 Alive (85%)
12	**Hosalkar et al. – 2007**[29]	Case series – IV	SAE	9 / 8 (88.9%)	27 (19 – 56)	9 Sacral (100%)	9 Primary (100%)	1 SpI (11.1%)	8 (88.9%)	7 PR (77.8%), 2 PD (22.2%)	2 (22.2%)	18.0 (6.0 – 30.0)	94.0 (46.0 – 254.0)	8 Alive (88.9%)
13	**Nakanishi et al. – 2013**[30]	Case series – IV	SAE	4 / 2 (50%)	36 (30 – 68)	4 Sacral (100%)	2 Primary (50%), 2 Recurrent (50%)	1 N (25%)	3 (75%)	3 PR (75%), 1 PD (25%)	0 (0%)	N/A	78.5 (14.0 – 141.0)	3 Alive (75%)
14	**Goldschlager et al. – 2015**[31]	Case series – IV	D	5 / 3 (100%)	33 (22 – 58)	2 T-L (40%), 1 Cervical (20%), 1 Lumbar (20%), 1 Sacral (20%)	5 Primary (100%)	0 (0%)	5 (100%)	4 PR (80%), 1 CR (20%)	0 (0%)	N/A	5.0 (4.0 – 26.0)	5 Alive (100%)
15	**Boriani et al. – 2019**[31]	Case series – IV	D	5 / 3 (60%)	37 (12 – 64)	3 Cervical (60%), 2 Sacral (40%)	3 Recurrent (60%), 2 Primary (40%)	0 (0%)	5 (100%)	5 PR (100%)	1 (25%)	15.0	57.0 (12.0 – 88.0)	5 Alive (100%)
16	**Sambri et al. – 2021**[31]	Case series – IV	D	10 / 5 (50%)	47.5 (32 – 73)	10 Sacral (100%)	8 Primary (80%), 2 Recurrent (20%)	0 (0%)	9 (90%)	9 PR (90%), 1 PD (10%)	1 (25%)	2.0	48.0 (8.5 – 102.0)	5 Alive (100%)
17	**Bukata et al. – 2021**[31]	Single-arm phase II trial – IIb	D	108 / 70 (64.8%)	32 (13 – 83)	67 Sacral (62%), 19 Thoracic (17.6%), 13 Cervical (12%), 9 Lumbar (8.3%)	62 Primary (57.4%), 46 Recurrent (42.6%)	10 ORN (9.3%), 4 NM (3.7%), 1 BF (0.9%), 1 HC (0.9%)	90 (83.3%)	55 PD (50.9%), 53 PR (49.1%)	12 (11.1%)	N/A	72.0 (41.0 – 81.0)	107 Alive (99.1%)

Abbreviations: **BF**, Bone fracture; **CR**, Complete response; **C-T**, Cervical-Thoracic; **D**, Denosumab; **DWH**, Delayed wound healing; **HC**, hypercalcemia; **L-S**, Lumbar-sacral; **MI**, Motor impairment; **N**, Neuropathy; **N/A**, Not available; **NM**, New malignancy; **ORN**, Osteoradionecrosis; **OS**, Overall survival; **PFS**, Progression free survival; **PD**, Progression disease; **PR**, Partial response; **RT**, Radiotherapy; **SAE**, Selective arterial embolization; **SD**, Stable disease; **SF**, Subcutaneous fibrosis; **SGCT**, Spine giant cell tumor; **SI**, Sensory impairment; **SN**, Skin necrosis; **SpI**, Spine instability; **SSP**, Severe sacral pain.

Supplementary File S2. Risk of bias assessments for included studies.

**Joanna Briggs Institute Checklist for Case Series – Criteria**
1. Were there clear criteria for inclusion in the case series?
2. Was the condition measured in a standard, reliable way for all participants included in the case series?
3. Were valid methods used for identification of the condition for all participants included in the case series?
4. Did the case series have consecutive inclusion of participants?
5. Did the case series have complete inclusion of participants?
6. Was there clear reporting of the demographics of the participants in the study?
7. Was there clear reporting of clinical information of the participants?
8. Were the outcomes or follow up results of cases clearly reported?
9. Was there clear reporting of the presenting site(s)/clinic(s) demographic information?
10. Was statistical analysis appropriate?
**Responses Options**: Yes, No, Unclear, Not Applicable (NA)
**Quality Rating**: Poor 0 – 3; Fair 4 – 7; Good 8 – 10

Study	1	2	3	4	5	6	7	8	9	10	Rating
Chuang et al. – 1981[33]	Yes	Yes	Yes	Yes	Yes	Yes	Yes	Yes	No	NA	8 – Good
Seider et al. – 1986[34]	Yes	Yes	Yes	Yes	Yes	Yes	Yes	Yes	No	NA	8 – Good
Schwartz et al. – 1989[35]	Yes	Yes	Yes	Yes	Yes	Yes	Yes	Yes	No	NA	8 – Good
Bennet et al. – 1993[36]	Yes	Yes	Yes	Yes	Yes	Yes	Yes	Yes	Yes	NA	9 – Good
Turcotte et al. – 1993[37]	Yes	Yes	Yes	Yes	Yes	Yes	Yes	Yes	No	NA	8 – Good
Hug et al. – 1995[38]	Yes	Yes	Yes	Yes	Yes	Yes	Yes	Yes	Yes	Yes	10 – Good
Malone et al. – 1995[26]	Yes	Yes	Yes	Yes	Yes	Yes	Yes	Yes	Yes	NA	9 – Good
Nair et al. – 1999[9]	Yes	Yes	Yes	Yes	Yes	Yes	Yes	Yes	No	NA	8 – Good
Lackman et al. – 2002[27]	Yes	Yes	Yes	Yes	Yes	Yes	Yes	Yes	No	NA	8 – Good
Lin et al. – 2002[11]	Yes	Yes	Yes	Yes	Yes	Yes	Yes	Yes	Yes	Yes	10 – Good
Caudell et al. – 2003[28]	Yes	Yes	Yes	Yes	Yes	Yes	Yes	Yes	No	NA	8 – Good
Hosalkar et al. – 2007[29]	Yes	Yes	Yes	Yes	Yes	Yes	Yes	Yes	Yes	NA	9 – Good
Nakanishi et al. – 2013[30]	Yes	Yes	Yes	Yes	Yes	Yes	Yes	Yes	No	NA	8 – Good
Goldschlager et al. – 2015[31]	Yes	Yes	Yes	Yes	Yes	Yes	Yes	Yes	No	NA	8 – Good
Boriani et al. – 2019[12]	Yes	Yes	Yes	Yes	Yes	Yes	Yes	Yes	No	NA	8 – Good
Sambri et al. – 2021[32]	Yes	Yes	Yes	Yes	Yes	Yes	Yes	Yes	No	Yes	9 – Good

**Joanna Briggs Institute Checklist for Clinical Trials – Criteria**
1. Was true randomization used for assignment of participants to treatment groups?
2. Was allocation to treatment groups concealed?
3. Were treatment groups similar at the baseline?
4. Were participants blind to treatment assignment?
5. Were those delivering treatment blind to treatment assignment?
6. Were outcomes assessors blind to treatment assignment?
7. Were treatment groups treated identically other than the intervention of interest?
8. Was follow up complete and if not, were differences between groups in terms of their follow up adequately described and analyzed?
9. Were participants analyzed in the groups to which they were randomized?
10. Were outcomes measured in the same way for treatment groups?
11. Were outcomes measured in a reliable way?
12. Was appropriate statistical analysis used?
13. Was the trial design appropriate, and any deviations from the standard RCT design (individual randomization, parallel groups) accounted for in the conduct and analysis of the trial?
**Responses Options**: Yes, No, Unclear, Not Applicable (NA)
**Quality Rating**: Poor 0 – 4; Fair 5 – 9; Good 10 – 13

Study	1	2	3	4	5	6	7	8	9	10	11	12	13	Appraisal
Bukata et al. – 2021[14]	NA	NA	NA	NA	NA	NA	NA	Yes	NA	NA	Yes	Yes	Yes	4 (see *comment*)

Comment: Despite the low score (4), the study was appraised with "good quality", and the risk of bias was considered low in regards of its design (i.e., open-label, single-arm, phase II trial)

Study	1	2	3	4	5	6	7	8	9	10	11	12	13	Appraisal
Bukata et al. – 2021[14]	NA	NA	NA	NA	NA	NA	NA	Yes	NA	NA	Yes	Yes	Yes	4 (see *comment*)

Comment: Despite the low score (4), the study was appraised with "good quality", and the risk of bias was considered low in regards of its design (i.e., open-label, single-arm, phase II trial)

Supplementary File S3. Funnel plots for indirect comparisons between denosumab vs radiotherapy vs selective arterial embolization for giant cell tumor of the spine: **A**. symptom improvement; **B**. positive radiological response; **C**. severe complications; **D**. local recurrence; **E**. distant metastases; overall survival at **F**. 6 months, **G**. 12 months, **H**. 18 months, and **I**. 24 months.

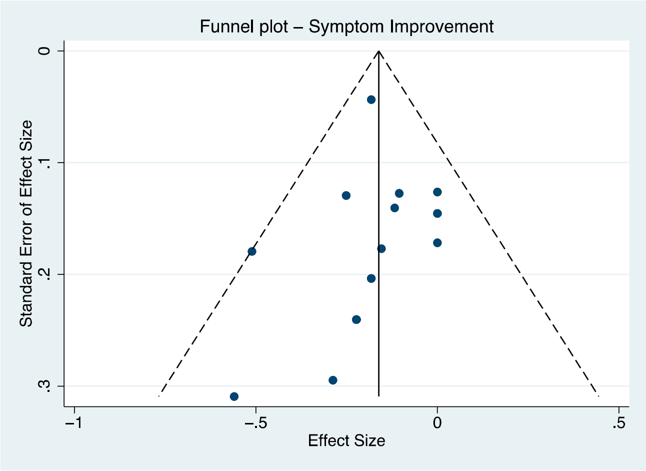

(A)

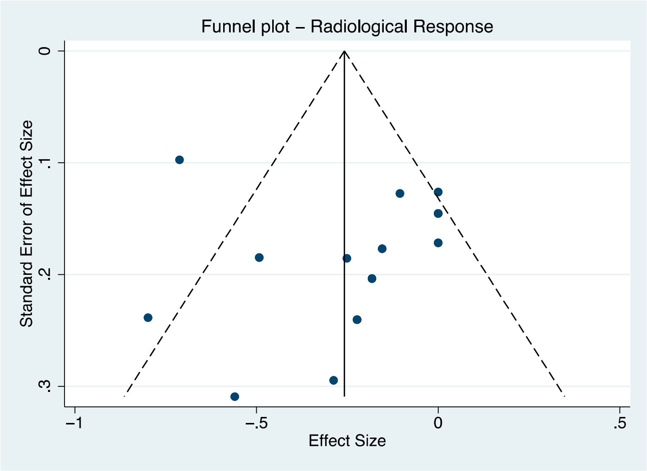

(B)

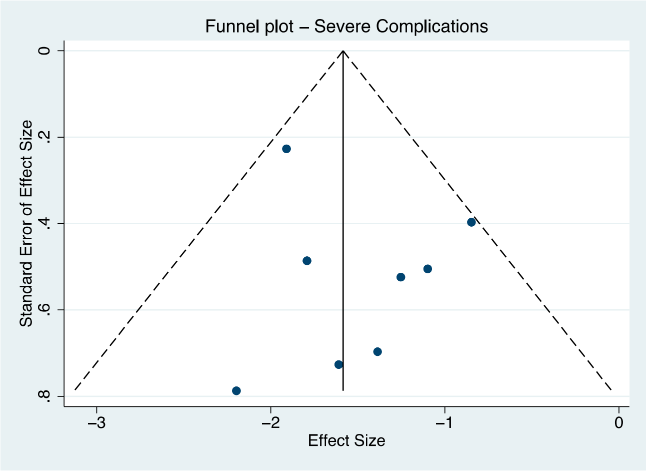

(C)

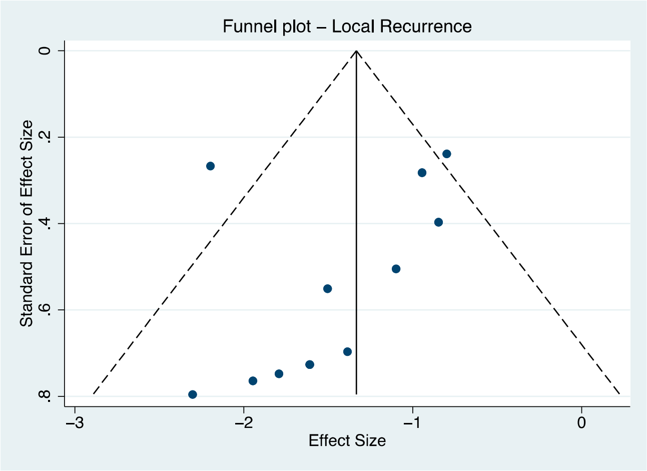

(D)

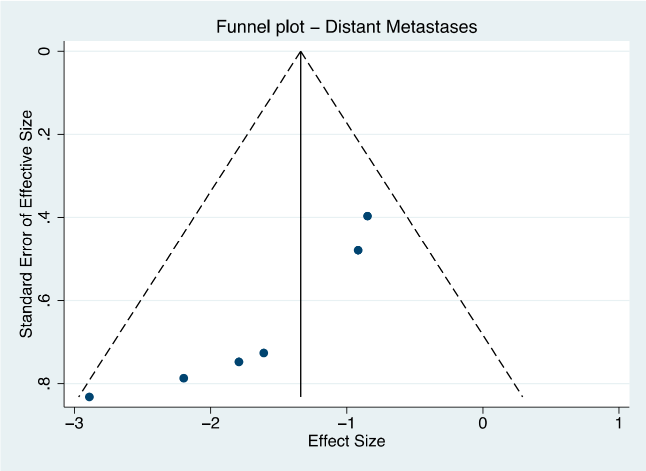
Funnel plot – Distant Metastases
Standard Error of Effective Size
0
.2
.4
.6
.8
−3
−2
−1
0
1
Effect Size

(E)

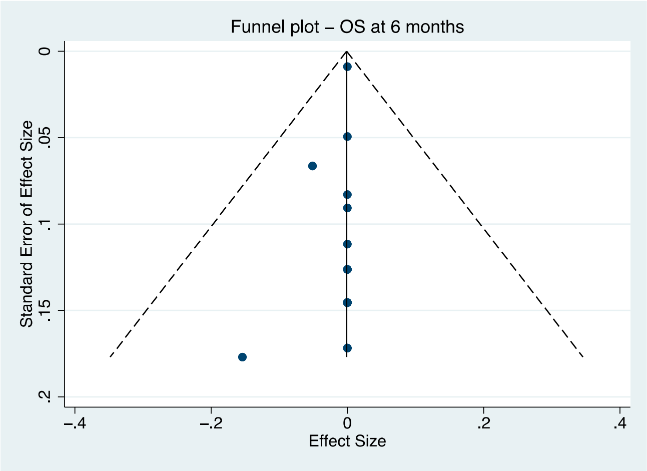
Funnel plot – OS at 6 months
Standard Error of Effect Size
0
.05
.1
.15
.2
−.4
−.2
0
.2
.4
Effect Size

(F)

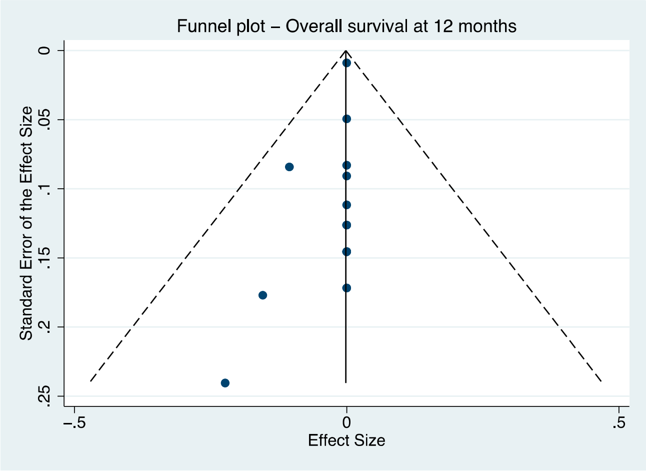
Funnel plot – Overall survival at 12 months
Standard Error of the Effect Size
0
.05
.1
.15
.2
.25
−.5
0
.5
Effect Size

(G)

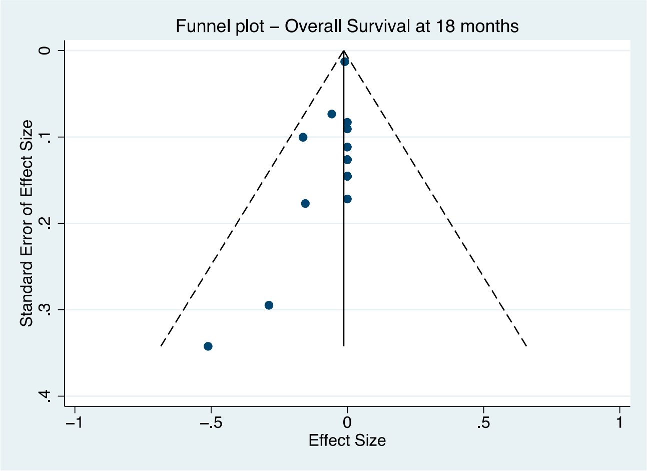
Funnel plot – Overall Survival at 18 months
Standard Error of Effect Size
0
.1
.2
.3
.4
−1
−.5
0
.5
1
Effect Size

(H)

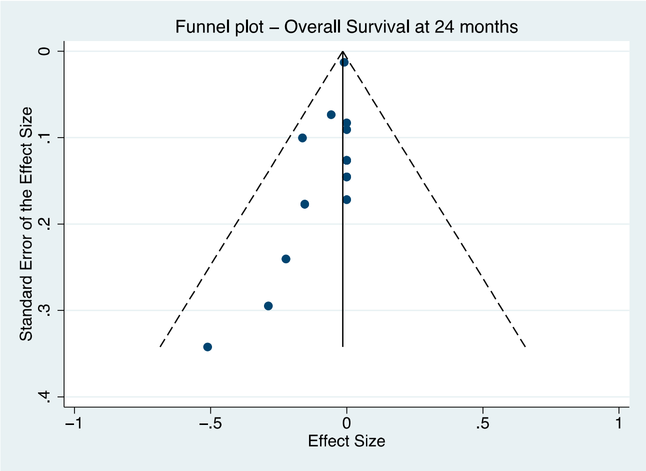
Funnel plot – Overall Survival at 24 months
Standard Error of the Effect Size
0
.1
.2
.3
.4
–1
–.5
0
.5
1
Effect Size

(I)

Supplementary File S4. Individual **f**orest plots for indirect comparisons between denosumab vs radiotherapy vs selective arterial embolization for giant cell tumor of the spine: **A**. symptom improvement; **B**. positive radiological response; **C**. severe complications; **D**. local recurrence; **E**. distant metastases; overall survival at **F**. 6 months, **G**. 12 months, **H**. 18 months, and **I**. 24 months.

Abbreviations: CI, confidence Interval; ES, Effect Size.

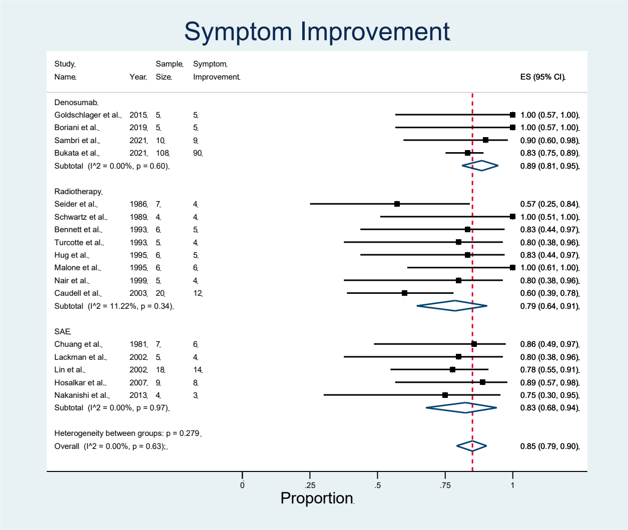

(A)

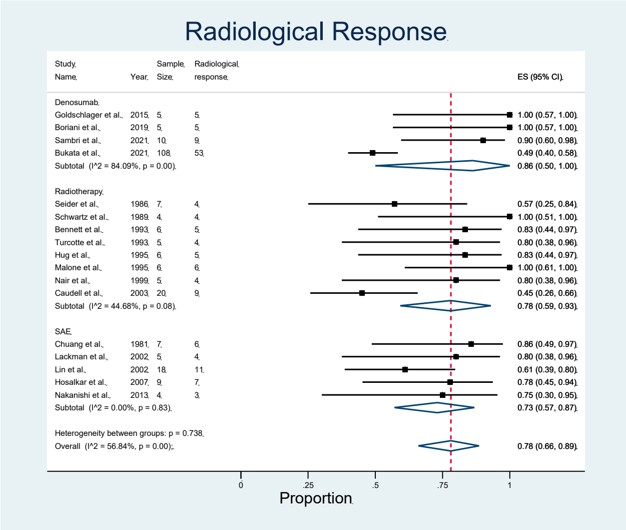

(B)

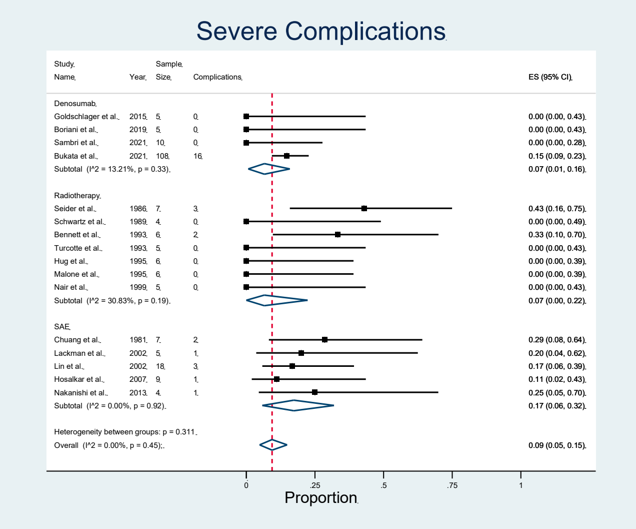

Severe Complications
Study Name | Year | Sample Size | Complications | ES (95% CI)
Denosumab
Goldschlager et al. 2015 5 0 0.00 (0.00, 0.43)
Boriani et al. 2019 5 0 0.00 (0.00, 0.43)
Sambri et al. 2021 10 0 0.00 (0.00, 0.28)
Bukata et al. 2021 108 16 0.15 (0.09, 0.23)
Subtotal (I^2 = 13.21%, p = 0.33) 0.07 (0.01, 0.16)
Radiotherapy
Seider et al. 1986 7 3 0.43 (0.16, 0.75)
Schwartz et al. 1989 4 0 0.00 (0.00, 0.49)
Bennett et al. 1993 6 2 0.33 (0.10, 0.70)
Turcotte et al. 1993 5 0 0.00 (0.00, 0.43)
Hug et al. 1995 6 0 0.00 (0.00, 0.39)
Malone et al. 1995 6 0 0.00 (0.00, 0.39)
Nair et al. 1999 5 0 0.00 (0.00, 0.43)
Subtotal (I^2 = 30.83%, p = 0.19) 0.07 (0.00, 0.22)
SAE
Chuang et al. 1981 7 2 0.29 (0.08, 0.64)
Lackman et al. 2002 5 1 0.20 (0.04, 0.62)
Lin et al. 2002 18 3 0.17 (0.06, 0.39)
Hosalkar et al. 2007 9 1 0.11 (0.02, 0.43)
Nakanishi et al. 2013 4 1 0.25 (0.05, 0.70)
Subtotal (I^2 = 0.00%, p = 0.92) 0.17 (0.06, 0.32)
Heterogeneity between groups: p = 0.311
Overall (I^2 = 0.00%, p = 0.45); 0.09 (0.05, 0.15)
0 .25 .5 .75 1
Proportion

(C)

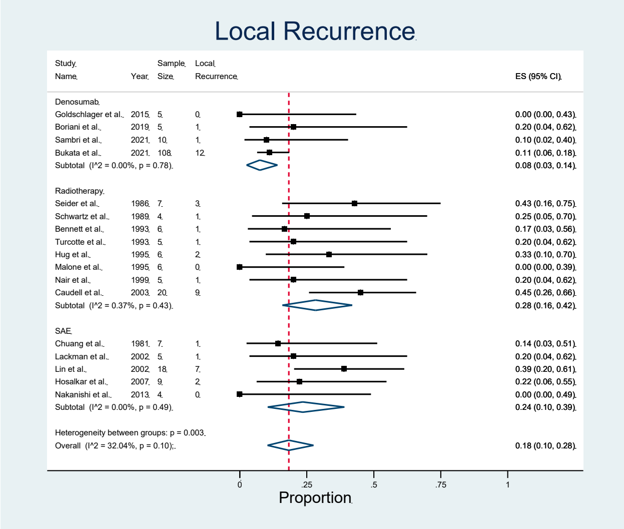

Local Recurrence
Study Name | Year | Sample Size | Local Recurrence | ES (95% CI)
Denosumab
Goldschlager et al. 2015 5 0 0.00 (0.00, 0.43)
Boriani et al. 2019 5 1 0.20 (0.04, 0.62)
Sambri et al. 2021 10 1 0.10 (0.02, 0.40)
Bukata et al. 2021 108 12 0.11 (0.06, 0.18)
Subtotal (I^2 = 0.00%, p = 0.78) 0.08 (0.03, 0.14)
Radiotherapy
Seider et al. 1986 7 3 0.43 (0.16, 0.75)
Schwartz et al. 1989 4 1 0.25 (0.05, 0.70)
Bennett et al. 1993 6 1 0.17 (0.03, 0.56)
Turcotte et al. 1993 5 1 0.20 (0.04, 0.62)
Hug et al. 1995 6 2 0.33 (0.10, 0.70)
Malone et al. 1995 6 0 0.00 (0.00, 0.39)
Nair et al. 1999 5 1 0.20 (0.04, 0.62)
Caudell et al. 2003 20 9 0.45 (0.26, 0.66)
Subtotal (I^2 = 0.37%, p = 0.43) 0.28 (0.16, 0.42)
SAE
Chuang et al. 1981 7 1 0.14 (0.03, 0.51)
Lackman et al. 2002 5 1 0.20 (0.04, 0.62)
Lin et al. 2002 18 7 0.39 (0.20, 0.61)
Hosalkar et al. 2007 9 2 0.22 (0.06, 0.55)
Nakanishi et al. 2013 4 0 0.00 (0.00, 0.49)
Subtotal (I^2 = 0.00%, p = 0.49) 0.24 (0.10, 0.39)
Heterogeneity between groups: p = 0.003
Overall (I^2 = 32.04%, p = 0.10); 0.18 (0.10, 0.28)
0 .25 .5 .75 1
Proportion

(D)

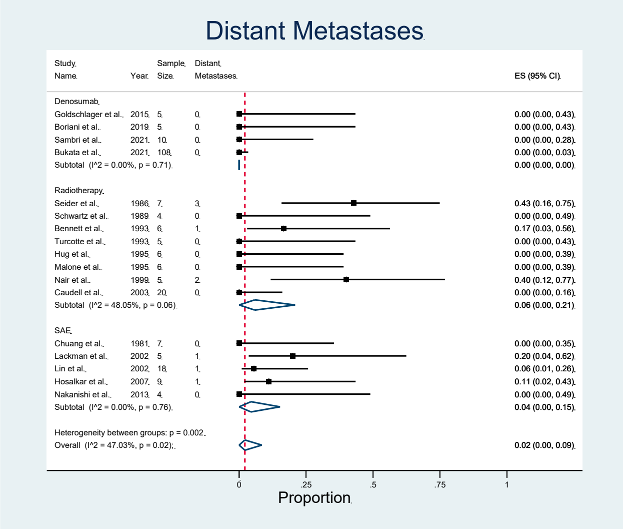

Distant Metastases
Study Name | Year | Sample Size | Distant Metastases | ES (95% CI)
Denosumab
Goldschlager et al. 2015 5 0 0.00 (0.00, 0.43)
Boriani et al. 2019 5 0 0.00 (0.00, 0.43)
Sambri et al. 2021 10 0 0.00 (0.00, 0.28)
Bukata et al. 2021 108 0 0.00 (0.00, 0.03)
Subtotal (I^2 = 0.00%, p = 0.71) 0.00 (0.00, 0.00)
Radiotherapy
Seider et al. 1986 7 3 0.43 (0.16, 0.75)
Schwartz et al. 1989 4 0 0.00 (0.00, 0.49)
Bennett et al. 1993 6 1 0.17 (0.03, 0.56)
Turcotte et al. 1993 5 0 0.00 (0.00, 0.43)
Hug et al. 1995 6 0 0.00 (0.00, 0.39)
Malone et al. 1995 6 0 0.00 (0.00, 0.39)
Nair et al. 1999 5 2 0.40 (0.12, 0.77)
Caudell et al. 2003 20 0 0.00 (0.00, 0.16)
Subtotal (I^2 = 48.05%, p = 0.06) 0.06 (0.00, 0.21)
SAE
Chuang et al. 1981 7 0 0.00 (0.00, 0.35)
Lackman et al. 2002 5 1 0.20 (0.04, 0.62)
Lin et al. 2002 18 1 0.06 (0.01, 0.26)
Hosalkar et al. 2007 9 1 0.11 (0.02, 0.43)
Nakanishi et al. 2013 4 0 0.00 (0.00, 0.49)
Subtotal (I^2 = 0.00%, p = 0.76) 0.04 (0.00, 0.15)
Heterogeneity between groups: p = 0.002
Overall (I^2 = 47.03%, p = 0.02); 0.02 (0.00, 0.09)
0 .25 .5 .75 1
Proportion

(E)

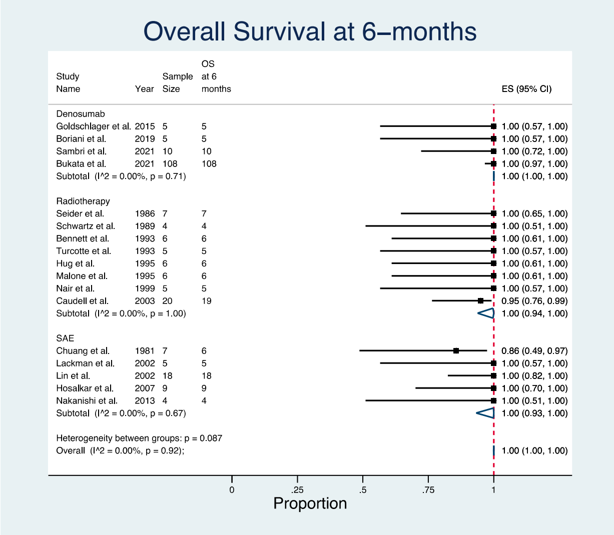

Overall Survival at 6–months
Study Name | Year | Sample Size | OS at 6 months | ES (95% CI)
Denosumab
Goldschlager et al. 2015 5 5 1.00 (0.57, 1.00)
Boriani et al. 2019 5 5 1.00 (0.57, 1.00)
Sambri et al. 2021 10 10 1.00 (0.72, 1.00)
Bukata et al. 2021 108 108 1.00 (0.97, 1.00)
Subtotal (I^2 = 0.00%, p = 0.71) 1.00 (1.00, 1.00)
Radiotherapy
Seider et al. 1986 7 7 1.00 (0.65, 1.00)
Schwartz et al. 1989 4 4 1.00 (0.51, 1.00)
Bennett et al. 1993 6 6 1.00 (0.61, 1.00)
Turcotte et al. 1993 5 5 1.00 (0.57, 1.00)
Hug et al. 1995 6 6 1.00 (0.61, 1.00)
Malone et al. 1995 6 6 1.00 (0.61, 1.00)
Nair et al. 1999 5 5 1.00 (0.57, 1.00)
Caudell et al. 2003 20 19 0.95 (0.76, 0.99)
Subtotal (I^2 = 0.00%, p = 1.00) 1.00 (0.94, 1.00)
SAE
Chuang et al. 1981 7 6 0.86 (0.49, 0.97)
Lackman et al. 2002 5 5 1.00 (0.57, 1.00)
Lin et al. 2002 18 18 1.00 (0.82, 1.00)
Hosalkar et al. 2007 9 9 1.00 (0.70, 1.00)
Nakanishi et al. 2013 4 4 1.00 (0.51, 1.00)
Subtotal (I^2 = 0.00%, p = 0.67) 1.00 (0.93, 1.00)
Heterogeneity between groups: p = 0.087
Overall (I^2 = 0.00%, p = 0.92); 1.00 (1.00, 1.00)
0 .25 .5 .75 1
Proportion

(F)

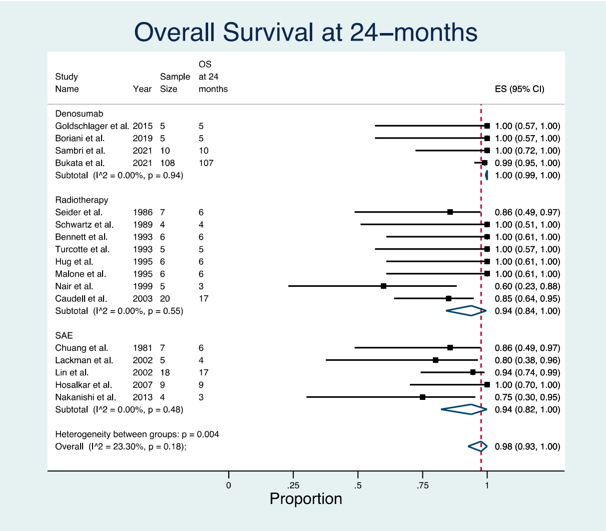
Overall Survival at 24–months
Study Name
Year
Sample Size
OS at 24 months
ES (95% CI)
Denosumab
Goldschlager et al. 2015 5 5 1.00 (0.57, 1.00)
Boriani et al. 2019 5 5 1.00 (0.57, 1.00)
Sambri et al. 2021 10 10 1.00 (0.72, 1.00)
Bukata et al. 2021 108 107 0.99 (0.95, 1.00)
Subtotal (I^2 = 0.00%, p = 0.94) 1.00 (0.99, 1.00)
Radiotherapy
Seider et al. 1986 7 6 0.86 (0.49, 0.97)
Schwartz et al. 1989 4 4 1.00 (0.51, 1.00)
Bennett et al. 1993 6 6 1.00 (0.61, 1.00)
Turcotte et al. 1993 5 5 1.00 (0.57, 1.00)
Hug et al. 1995 6 6 1.00 (0.61, 1.00)
Malone et al. 1995 6 6 1.00 (0.61, 1.00)
Nair et al. 1999 5 3 0.60 (0.23, 0.88)
Caudell et al. 2003 20 17 0.85 (0.64, 0.95)
Subtotal (I^2 = 0.00%, p = 0.55) 0.94 (0.84, 1.00)
SAE
Chuang et al. 1981 7 6 0.86 (0.49, 0.97)
Lackman et al. 2002 5 4 0.80 (0.38, 0.96)
Lin et al. 2002 18 17 0.94 (0.74, 0.99)
Hosalkar et al. 2007 9 9 1.00 (0.70, 1.00)
Nakanishi et al. 2013 4 3 0.75 (0.30, 0.95)
Subtotal (I^2 = 0.00%, p = 0.48) 0.94 (0.82, 1.00)
Heterogeneity between groups: p = 0.004
Overall (I^2 = 23.30%, p = 0.18); 0.98 (0.93, 1.00)
0 .25 .5 .75 1
Proportion

(G)

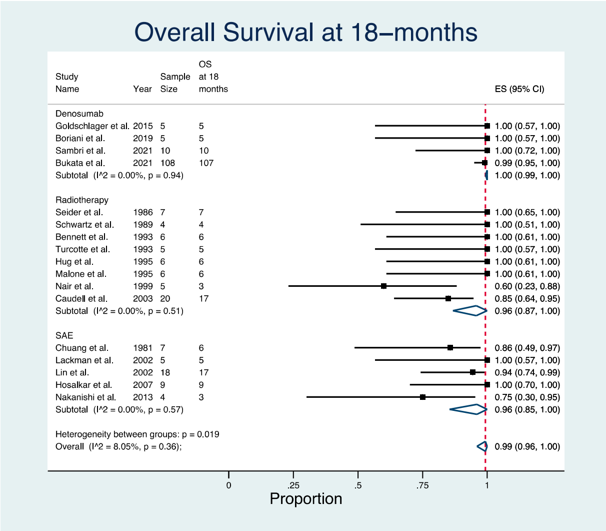
Overall Survival at 18–months
Study Name
Year
Sample Size
OS at 18 months
ES (95% CI)
Denosumab
Goldschlager et al. 2015 5 5 1.00 (0.57, 1.00)
Boriani et al. 2019 5 5 1.00 (0.57, 1.00)
Sambri et al. 2021 10 10 1.00 (0.72, 1.00)
Bukata et al. 2021 108 107 0.99 (0.95, 1.00)
Subtotal (I^2 = 0.00%, p = 0.94) 1.00 (0.99, 1.00)
Radiotherapy
Seider et al. 1986 7 7 1.00 (0.65, 1.00)
Schwartz et al. 1989 4 4 1.00 (0.51, 1.00)
Bennett et al. 1993 6 6 1.00 (0.61, 1.00)
Turcotte et al. 1993 5 5 1.00 (0.57, 1.00)
Hug et al. 1995 6 6 1.00 (0.61, 1.00)
Malone et al. 1995 6 6 1.00 (0.61, 1.00)
Nair et al. 1999 5 3 0.60 (0.23, 0.88)
Caudell et al. 2003 20 17 0.85 (0.64, 0.95)
Subtotal (I^2 = 0.00%, p = 0.51) 0.96 (0.87, 1.00)
SAE
Chuang et al. 1981 7 6 0.86 (0.49, 0.97)
Lackman et al. 2002 5 5 1.00 (0.57, 1.00)
Lin et al. 2002 18 17 0.94 (0.74, 0.99)
Hosalkar et al. 2007 9 9 1.00 (0.70, 1.00)
Nakanishi et al. 2013 4 3 0.75 (0.30, 0.95)
Subtotal (I^2 = 0.00%, p = 0.57) 0.96 (0.85, 1.00)
Heterogeneity between groups: p = 0.019
Overall (I^2 = 8.05%, p = 0.36); 0.99 (0.96, 1.00)
0 .25 .5 .75 1
Proportion

(H)
